# Optimised Protocol for Managing Failed Catheterisation: Leveraging Bedside Retrograde Urethrography and Cystourethroscopy

**DOI:** 10.7759/cureus.69363

**Published:** 2024-09-13

**Authors:** Basavesh S Patil, Siddanagouda B Patil, Vinay S Kundargi, Santosh R Patil, Manoj K Vaidya, Vikas Shukla

**Affiliations:** 1 Urology, Shri B.M. Patil Medical College Hospital and Research Centre, Bijapur Lingayat District Educational (BLDE) Association (Deemed to be University), Vijayapura, IND

**Keywords:** cystourethroscopy, retrograde urethrography, suprapubic catheterisation, urethral catheterisation, urethral injury

## Abstract

Introduction

Urethral catheterization is a routine procedure often required for many hospitalized patients. Various conditions, such as meatal stenosis, stricture urethra, false passage, benign prostatic hyperplasia, bladder neck contractures, and impacted urethral stones, can contribute to difficulty in catheterisation. In the setting of failed attempts at per urethral catheter placement, the subsequent intervention is suprapubic catheter (SPC) insertion. SPC placement has its associated complications and causes inconvenience to the patients. We framed an algorithm to minimise the need for SPC insertion in cases of difficult per urethral catheterisation in a non-trauma setting. This study aimed to evaluate the common causes of difficult per urethral catheterisation and establish the efficacy of our algorithm in managing difficult catheterisation with bedside retrograde urethrography (RGU) and cystoscopy while avoiding SPC placement.

Materials and methods

This prospective observational study was conducted from September 2022 to June 2024. Patients admitted with urinary retention or requiring routine catheterisation, with one failed attempt at catheterisation, were included in the study. Our algorithm for the management of difficult catheterisation in a non-trauma setting, to avoid SPC, integrates a bedside RGU and retrograde urethroscopy using either a 15.5 Fr cystoscope sheath or a 6 Fr ureteroscope to identify the urethral pathology, followed by dilatation and per urethral catheterisation.

Results

Among 55 patients (aged 34-82 years), 48 (87.27%) were male and seven (12.73%) were female. The most common indication for catheterisation was routine catheterisation for output monitoring (n = 30; 54.54%), followed by acute retention (n = 25; 45.45%). Bulbar urethral stricture (n = 28; 50.9%) was the most common cause of difficult catheterisation, followed by meatal/sub-meatal narrowing (n = 13; 23.63%), enlarged prostate or high bladder neck (n = 4; 7.27%), and impacted stones (n = 3; 5.45%). Successful catheterisation was achieved in 48 male patients following urethroscopy with a 6 Fr ureteroscope or 15.5 Fr cystoscope. In females, reducing the pelvic organ prolapse enabled catheterisation in two cases, while five required serial dilatation and catheterisation. Successful per-urethral catheterisation was achieved in all 55 (100%) patients, thus avoiding SPC.

Conclusions

Conventional blind catheterisation techniques have limited success in the setting of failed initial catheterisation. This approach, which employs bedside fluoroscopy and direct visualisation of the urethra using a cystoscope or ureteroscope, helped achieve higher success rates (n = 55; 100%) for difficult per-urethral catheterisation and avoided the need for SPC. Proper implementation of this protocol for dealing with difficult per-urethral catheterisation will reduce the unnecessary burden on the healthcare system by minimising the potential iatrogenic urethral injuries and reducing the need for SPC.

## Introduction

Urethral catheterisation is a routine medical procedure performed in both Outpatient Departments (OPDs) and hospitalised patients. Indwelling urinary tract catheters are often overused, and approximately 25% of the patients undergo catheterisation during their hospitalisation [1]. Difficult catheterisation is a common challenge faced by every urologist. Improper technique or a tight sphincter in an anxious individual may be the reason for difficult catheterisation in a patient with a normal urethra. The pathologic causes for difficult catheterisation include phimosis, meatal stenosis, stricture urethra, false passage, benign prostatic hyperplasia (BPH), and bladder neck contractures. Repeated unsuccessful attempts to catheterise these patients can cause discomfort and result in complications like bleeding, false passage, and urethral injury, which may lead to urethral strictures in the future [2]. In these situations, attempts to catheterise using a conventional blind technique may further cause urethral injury [3]. Improper insertion of catheters may cause urethral trauma or false passage, which may significantly contribute to morbidity and increase the length of hospital stay, as the patient may need secondary interventions and complex follow-up evaluations [4].

Once there is a failed attempt at catheterisation, the recommended practices are passage of guidewire under vision (using a flexible or rigid cystoscope) or blind passage of guidewire, followed by advancement of a modified urethral catheter immediately or after dilatation, and instilling 60 cc saline through the catheter while advancing it. With advancements in medical technology, specially designed Foley catheters with integrated hydrophilic guidewire have been devised to facilitate catheterisation [2,5]. All these techniques have variable results, and when they fail, the patient might ultimately need suprapubic catheterisation (SPC), which can have associated complications like infection, bleeding, bowel perforation, and bladder perforation [6]. To avoid additional interventions like SPC and other secondary procedures, we are proposing a new algorithm to manage difficult catheterisation by integrating a bedside retrograde urethrogram (RGU) and retrograde urethroscopy using either a 15.5 Fr cystoscope sheath or a 6 Fr ureteroscope to identify the urethral pathology, followed by dilatation and per urethral catheterisation.

## Materials and methods

**Figure 1:**
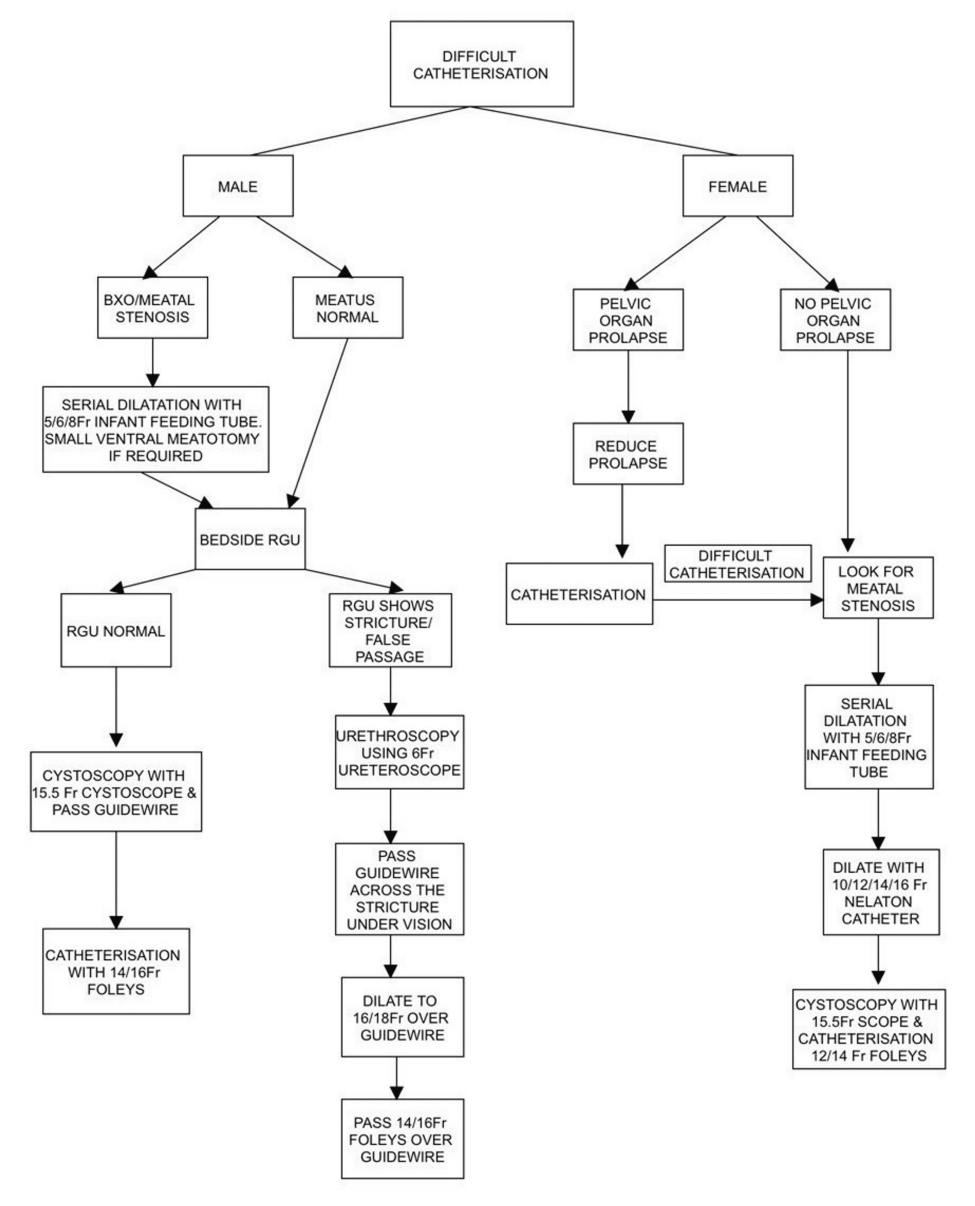
Algorithm for difficult catheterisation in non-trauma setting with bedside RGU and cystoscopy BXO: Balanitis xerotica obliterans; RGU: Retrograde urethrography

This prospective, single-centre, observational study was conducted from September 2022 to June 2024. We have proposed an algorithm for managing difficult catheterisation in a non-trauma setting (Figure 1), intending to avoid SPC. After obtaining clearance from the Institutional Ethical Committee (BLDE(DU)/IEC/1109/2024-25), we included all patients above 18 years of age presenting with urinary retention or hospitalised patients requiring catheterisation for output monitoring, who had one failed attempt to place a per urethral catheter. A trained resident doctor with adequate expertise attempted initial catheterisation with a 14/12 Fr Foley catheter after instilling 10 mL of xylocaine jelly. If negotiating a Foley catheter was not possible, an attempt was made to pass a 10 Fr infant feeding tube (IFT). This entire process was considered the 'first attempt to catheterise'. Inability to pass the Foley catheter or a 10 Fr IFT by the resident doctor during the initial attempt was considered a 'failed initial attempt at catheterisation,' and these patients met the criteria to be included in the study. Any further attempts at blind catheterisation were avoided to reduce the possibility of inadvertent injury to the urethra. We excluded patients who refused to participate in the study and opted for SPC, those with hip or pelvic deformities that could cause difficulty in giving a lithotomy position, and patients presenting with trauma to the perineum.

Anticipating a success rate of 87.2% for inserting the urethral catheter, as per a study by Jeong et al. [7], we arrived at the required sample size of 55 patients with a 95% level of confidence and 9% absolute precision. The data recorded are presented as means and percentages.

A brief history of urinary symptoms was taken either from the patient or the attendant. Patients were examined to check for fullness of the lower abdomen to confirm a distended bladder. The genital examination was performed to note the presence of phimosis, meatal stenosis, stricture, and obstructing urethral stone. A per rectal examination was conducted to check for prostate enlargement in males, and a per vaginal examination was performed in females to assess pelvic organ prolapse. Written informed consent was obtained from all patients after explaining the protocol of the proposed study.

In the male patients, bedside RGU was performed using urografin (76%) and a mobile X-ray unit with a 12 x 15 inch X-ray cassette to confirm the cause of obstruction. In patients with normal RGU and a large prostate or median lobe enlargement, cystoscopy was performed using a large 15.5 Fr sheath. Those who had an enlarged prostate or median lobe were catheterized using a 16 Fr Foley catheter after instilling 10 mL of xylocaine jelly.

**Figure 2:**
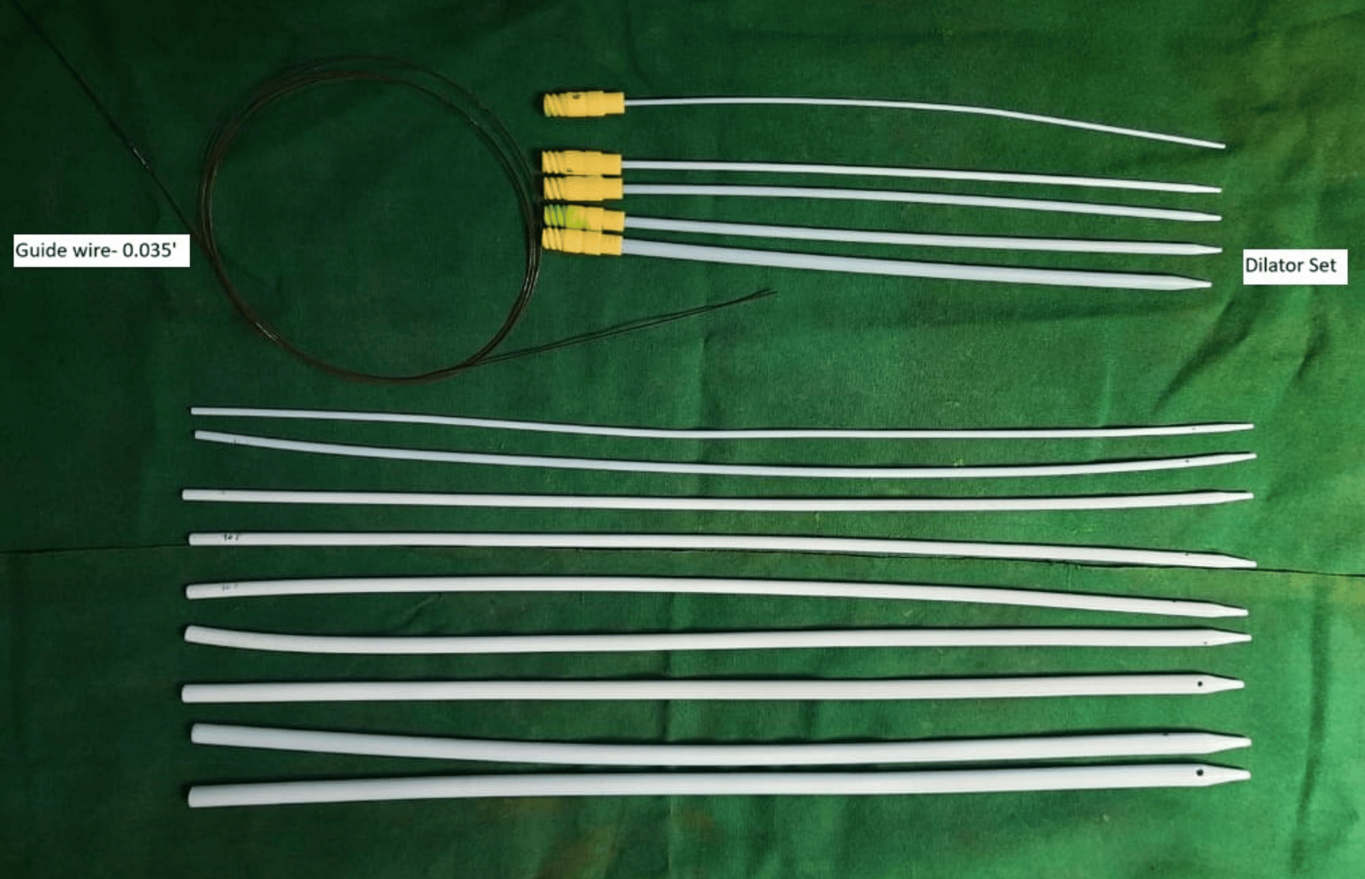
A 0.035 inch guidewire with dilators

**Figure 3:**
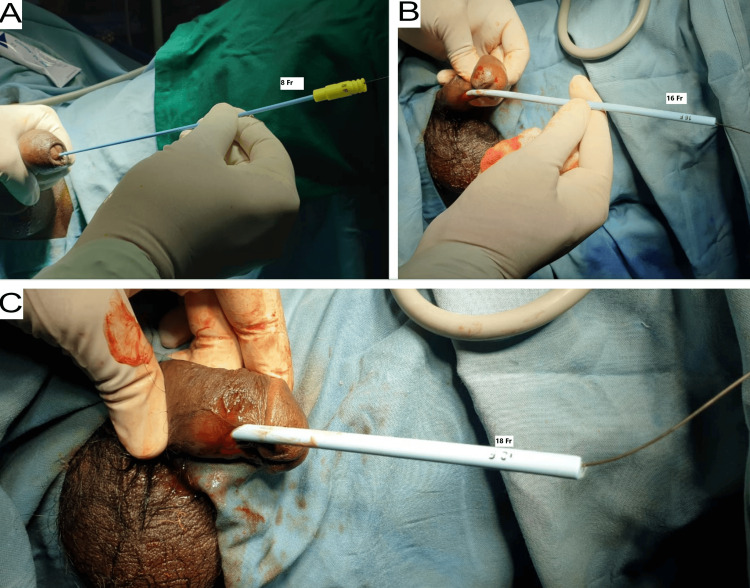
Serial dilatation of the urethra over guidewire (A) 8 Fr, (B) 16 Fr, and (C) 18 Fr

**Figure 4:**
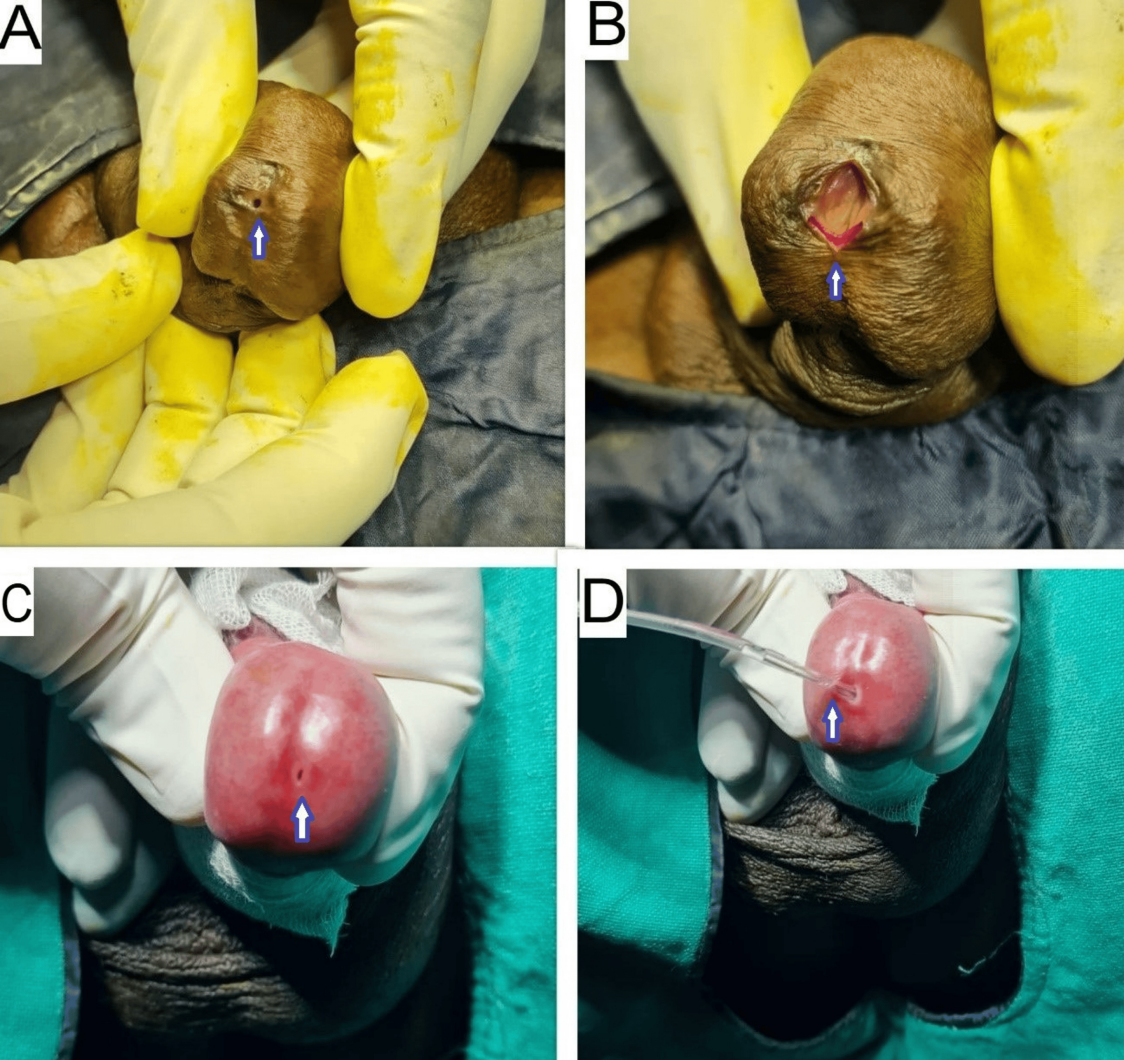
(A) Meatal stenosis; (B) small ventral meatotomy done to facilitate access to urethra depicted by blue arrow; (C)-(D) meatal stenosis and gentle dilatation with infant feeding tube

In patients with meatal stenosis and balanitis xerotica obliterans (BXO) changes on the glans penis, a RGU was performed using 5 Fr, 6 Fr, or 8 Fr IFT to confirm the site and length of the urethral stricture. Using a 6 Fr ureteroscope, the urethra was examined up to the site of the stricture. A 0.035-inch guidewire was passed across the stricture into the bladder; its position in the bladder was confirmed with an X-ray, and, over the guidewire, the urethra was dilated using filiform urethral dilators (6-18 Fr) (Figures 2-3). In cases where the meatus was very narrow, a small ventral meatotomy was performed (Figure 4). Later, a 14 Fr or 16 Fr Foley catheter was passed over the guidewire into the bladder.

In female patients with pelvic organ prolapse, catheterisation was attempted after reduction of the prolapse, and those who had meatal stenosis underwent dilatation using serial metal dilators and were catheterised.

Since the patients included in the study had one documented attempt of difficult or failed catheterisation, and keeping in view a differential diagnosis of stricture urethra or false passage, we have incorporated RGU into the algorithm. The other causes for difficult catheterisation could be an enlarged prostate or a high bladder neck, which cannot be definitively established with an RGU and would require a cystourethroscopy.

Further, although there are certain practical challenges in executing the bedside RGU, including radiation exposure, RGU is less invasive than cystourethroscopy or even blind attempts to catheterise following a failed initial attempt.

As one of the objectives of this study was to investigate the causes of difficult catheterisation in a hospital setting, and not merely per urethral catheter placement, we have incorporated RGU, followed by cystourethroscopy, into our study algorithm.

## Results

The total number of patients included in the study was 55. Patients in the study group were between 34 and 82 years of age, with 48 (87.27%) male patients and seven (12.7%) female patients (Table 1). The most common indication for catheterisation was routine catheterisation for output monitoring (n = 30; 54.54%), followed by acute retention (n = 25; 45.45%). The most common cause of difficult catheterisation was stricture in the bulbar urethra, measuring 0.5 to 1.5 cm, which was noted in 28 (50.90%) patients. A major fraction (n = 21; 38.18%) of the study participants had a prior history of urethral catheterisation. Furthermore, 13 patients (23.63%) had undergone some endourological procedure in the form of cystoscopy, dilatation, or visual internal urethrotomy in the past. Both these factors have possibly contributed to the high prevalence of strictures in our study.

**Table 1:** Table showing the patient parameters and findings of retrograde urethroscopy (RGU) and cystoscopy

Study parameters	Observations	n (%)
Gender	Female	7 (12.7%)
	Male	48 (87.27%)
	Grand total	55
Associated medical conditions	Renal failure/chronic kidney disease	7 (12.27%)
	Stroke/cerebrovascular accident	6 (10.90%)
	Diabetes mellitus	22 (40%)
	Hypertension	9 (16.36%)
	Hypertension + diabetes mellitus	5 (9.09%)
	Nil	6 (10.90%)
Previous history of catheterisation	No prior catheterisation	34 (61.82%)
	Prior catheterisation	21 (38.18%)
Indication for catheterisation	Acute retention	25 (45.45%)
	Routine catheterisation	30 (54.54%)
History of previous urologic intervention (cystoscopy, dilatation, visual internal urethrotomy)	No	42 (76.36%)
	Yes	13 (23.63%)
Meatal stenosis	Absent	37 (67.27%)
	Present	18 (32.72%)
Balanitis xerotica obliterans (BXO)	Absent	48 (87.27%)
	Present	7 (12.72%)
Retrograde urethroscopy (RGU) findings	Bulbar stricture	28 (50.90%)
	Impacted stone	3 (5.45%)
	Meatal stenosis	13 (23.63%)
	Normal	4 (7.27%)
	Not done	7 (12.72%)
Cystoscopy (using 15.5 Fr cystoscope or 6 Fr ureteroscope)	15.5 Fr sheath	6 (10.90%)
	6 Fr ureteroscope	42 (76.36%)
	Not done	7 (12.72%)
Final diagnosis/cause for difficult catheterisation after retrograde urethroscopy (RGU) and cystoscopy	Benign prostatic hyperplasia (BPH)	4 (7.27%)
	Meatal stenosis	13 (23.63)
	Impacted stone	2 (3.63%)
	Balanitis xerotica obliterans (BXO)	5 (9.09%)
	Prolapse	2 (3.63%)
	Prolapse with meatal stenosis	5 (9.09%)
	Stricture	23 (41.81%)
	Stricture with impacted stone	1 (1.81%)

In this study, 13 (23.63%) patients had meatal stenosis. Additionally, five patients (9.09%) had BXO. Following meatal dilatation, a 5 Fr/6 Fr/8 Fr IFT was used to conduct an RGU, which confirmed the stricture secondary to BXO.

**Figure 5:**
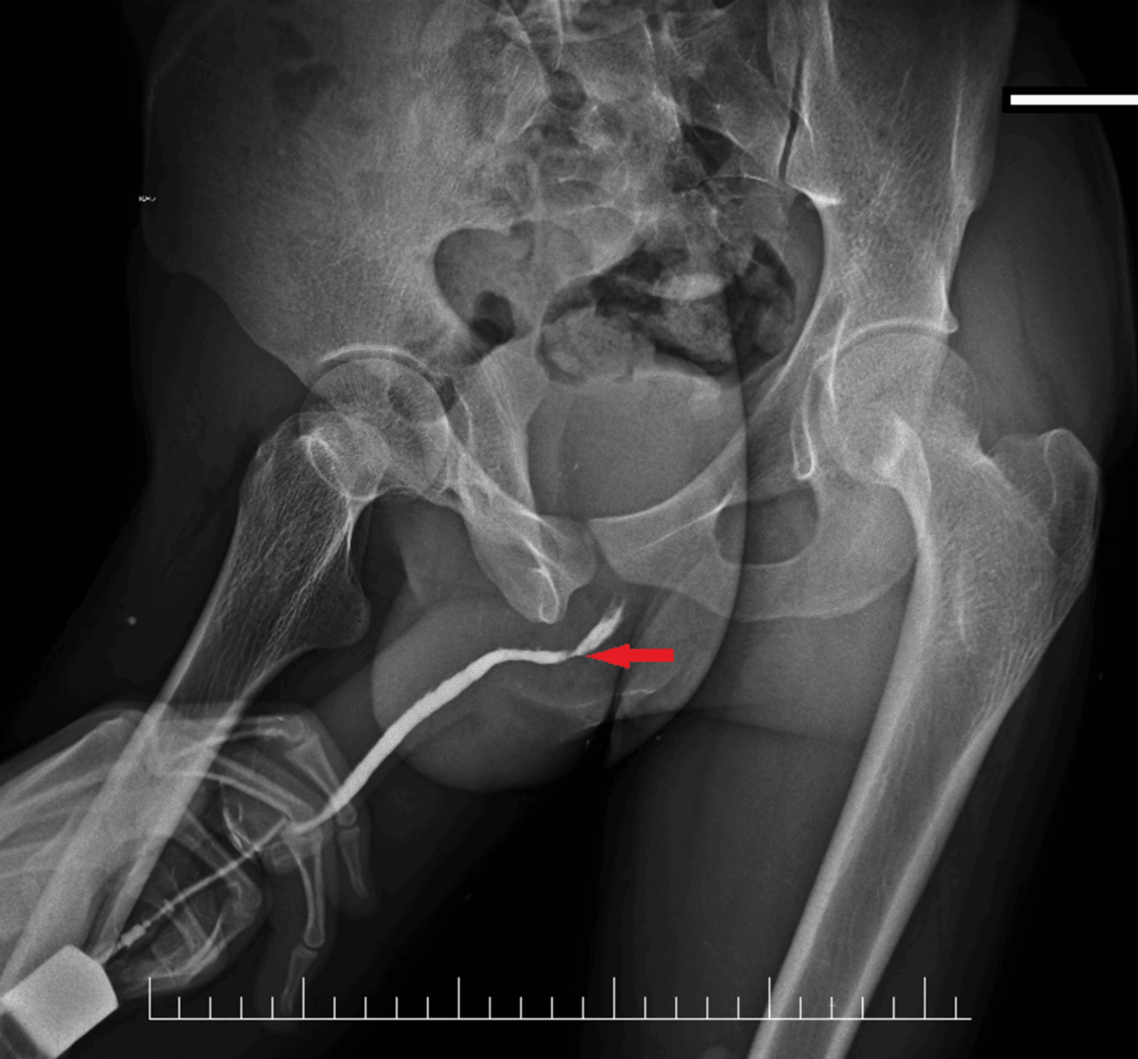
Bedside retrograde urethrography (RGU) film The red arrow marks the site of the bulbar urethral stricture.

All the 42 male patients who demonstrated stricture (Figure 5) on RGU - bulbar urethra stricture (n = 28; 50.90%), meatal/sub-meatal narrowing (n = 13; 23.63%), and one patient with stricture and an impacted urethral stone (n = 1; 1.81%) - underwent urethroscopy with a 6 Fr ureteroscope and passage of a 0.035” guidewire beyond the stricture into the bladder, which was confirmed using X-ray. This was followed by dilatation over a guidewire (6-18 Fr) and placement of a 14 Fr/16 Fr Foley catheter over the guidewire.

Four (7.27%) male patients who had normal RGU underwent cystoscopy using a 15.5 Fr sheath. Two (3.63%) patients had a large prostate with median lobe enlargement, and two (3.63%) had prostatomegaly with elevated bladder neck. They were catheterised with a 16 Fr Foley catheter. Two patients had impacted urethral stones, which did not permit catheter passage.

Among the seven female patients who had complete vaginal prolapse, two (3.63%) could be catheterised with a 14 Fr Foley catheter by simply reducing the prolapse. Five (9.09%) patients had meatal stenosis and required serial dilatation, followed by catheterisation with a 14 Fr/16 Fr Foley catheter. Successful per urethral catheterisation was achieved in all 55 patients, thereby avoiding any need for SPC.

Although this protocol offers a simplified and logical approach to dealing with failed attempts at catheterisation, it is associated with certain practical difficulties. As most of these patients, in the intensive care unit (ICU) setting, are critically ill, positioning them appropriately for the RGU is a demanding task. The image quality of a bedside RGU is inferior to that of a regular RGU and may pose diagnostic challenges. While performing the bedside RGU, the digital X-ray cassette needs to be sent back to the X-ray console to be placed in the image reader, which is time-consuming. This can be overcome by using C-arm image intensifiers along with C-arm-compatible beds. However, the availability of such a setup is very limited and cost-intensive.

## Discussion

In the non-trauma setting, the common indications for catheterisation are routine monitoring of urine output in critically ill patients and acute urinary retention [7]. In our study, we also noted that routine catheterisation for output monitoring (n = 30; 54.54%), followed by acute retention (n = 25; 45.45%), were the common indications for per urethral catheterisation.

Urinary catheterisation is a straightforward procedure when appropriately performed using strict aseptic precautions. Even though it is a simple procedure, it can sometimes result in complications like infection, bleeding, and trauma to the urethra. Injury to the urethra during catheterisation transforms a relatively simple procedure into an emergency, adding to the morbidity and cost of treatment, along with the need for prolonged follow-up.

Although an enlarged prostate or a luminal stricture contributes to difficult catheterisation, a vast majority of urological consultations for difficult catheterisation are due to urethral trauma that has occurred in a urethra with a non-constricted but altered anatomy, such as BPH, a tight external sphincter in young males, or a high bladder neck [2]. It is always advised that catheterisation be performed by trained medical staff to facilitate atraumatic passage and avoid the setting of a difficult catheterisation needing secondary procedures or intervention. Several studies have shown that adequate staff training regarding safe catheterisation practices reduces the chances of urethral catheterisation injury fivefold, from 3.2/1000 to 0.7/1000 [8].

As a second-line method for failed initial catheterisation, the safety and effectiveness of the Seldinger technique with a straight, hydrophilic guidewire have been well established and can be performed by non-urology-trained doctors in the bedside setting [9]. Few other authors have proposed the use of flexible cystoscopy and the passing of a guidewire under vision, followed by threading of the catheter down the guidewire [10]. When all these efforts fail, the only option left is SPC, which comes with its own pros and cons. Hence, there is a small window between a failed initial attempt at catheterisation and SPC insertion, during which timely and methodical intervention by a urologist can prevent urethral injury and, at the same time, obviate the need for an SPC.

Kim et al. have proposed a new technique of RGU-assisted urethral catheterisation that combines the Seldinger technique, fluoroscopy, and the application of hydrostatic pressure to the urethra. It has the benefit of delineating the urethral anatomy and confirming the position of the tip of the guidewire. They achieved 69.1% success in catheterising the patients with their RGU-assisted technique [11]. We have employed a similar, modified fluoroscopy technique using the bedside X-ray unit and cystoscopy with a 15.5 Fr sheath or a semirigid ureteroscopy, which helps to establish the anatomic defect, prevent further urethral injury, and facilitate dilatation and catheterisation, which may avoid secondary procedures and associated morbidity in most of the patients. With our proposed algorithm, we have been able to achieve a very high success rate (n = 55; 100%) in placing per urethral catheter and avoiding SPC in patients with failed attempts at initial catheterisation.

Willette et al. conducted a feasibility study of a protocol to manage difficult catheterisation using visually guided equipment. They used a special device with a 0.6 mm fibre optic bundle, placed inside a 14 Fr triple lumen catheter. The device had an irrigation port and could be connected to a camera to provide a real-time view. The success rate of catheterisation using this visually guided device was 100%, with the median number of passes required being only one. The procedure time was also less than 17 minutes in the majority of the cases [12]. This study further substantiates our observation that the RGU and cystourethroscopy-assisted protocol for placing the catheters has a high success rate.

Price and McKeon evaluated the effectiveness of a nurse-led difficult catheterisation program. Among 435 patients surveyed retrospectively, they reported a success rate of 92%. Strict adherence to their comprehensive program helped to improve the catheter insertion technique, reduced the urology consultations, and was effective in reducing catheter-associated urinary tract infections [13]. This shows that following a particular algorithm to manage difficult catheterisation, and proper training of the medical staff, can reduce iatrogenic urethral injuries and the need for additional urologic consultation.

Patel and Antil have described a technique to manage difficult catheterisation using a soft-tipped, hydrophilic guidewire to insert a three-way urethral catheter. They demonstrated a 100% success rate. Follow-up of these patients showed no complications or adverse effects [14]. This study, however, did not use any visual guidance, like fluoroscopy or cystoscopy, as was done in our study.

The algorithm proposed in our study is broadly applicable to the adult population. Unlike adult patients, catheterisation in children is performed for various diagnostic and therapeutic indications. In paediatric patients, to avoid urethral trauma and minimise complications, the selection of the proper size of the catheter, based on the child’s body weight and age, becomes important [15]. Hence, our algorithm cannot be directly applied to paediatric settings.

In the setting of traumatic urethral injury, RGU remains the initial diagnostic tool to assess the status of the urethra. Based on the presence of pelvic fracture and associated injuries, the management may vary from early operative repair or early endoscopic realignment to delayed urethroplasty, after suprapubic catheterisation [16]. Separate algorithms have to be developed for the management of traumatic urethral injuries.

Based on our experience with difficult catheterisation in hospitalised patients in non-trauma settings, we have designed a modified algorithm that can help ensure safe urethral catheterisation. This algorithm (Figure 1) is easy to implement and integrate into standard catheterisation protocols to manage difficult catheterisation and avoid inadvertent injury to the urethra. The advantages of the proposed algorithm are mentioned in Table 2.

**Table 2:** Advantages of the proposed algorithm

Advantages
Avoids the need for suprapubic catheterisation and its complications
Avoids secondary procedures after discharge (retrograde urethroscopy, cystoscopy)
Simplifies evaluation of the probable cause for retention of urine
Reduces the investigation and hospitalisation costs

This protocol offers to simplify the management of difficult catheterisation, but the study has certain limitations. The sample size of 55 is relatively small, and hence, to validate this protocol, larger studies need to be conducted. This protocol cannot be applied to strictures of longer lengths. The high success rate (100%) reported in this study may partly be attributable to the fact that the implementation of this protocol was done by trained urologists, whereas, in actual clinical practice, the management of difficult catheterisation may be done by trainee urologists or different cadres of healthcare professionals, which may have an impact on the overall success rate. Another major limitation of this study is the lack of long-term follow-up of the patients and the details regarding the recurrence of strictures or the need for secondary procedures to treat the strictures.

## Conclusions

Urologists routinely encounter cases of difficult urethral catheterisation. Catheterisation for output monitoring, followed by acute retention, were the most common indications for catheter placement in a hospital setting. Urethral stricture is the most common cause of difficult catheterisation in males, followed by meatal/sub-meatal narrowing, BXO, BPH, and impacted stones. In females, prolapse with meatal stenosis was the most commonly reported cause of difficult catheterisation. As blind catheterisation techniques have a limited success rate in the setting of failed initial catheterisation, this algorithm, with a modified technique employing bedside fluoroscopy and direct visualisation with a cystoscope or ureteroscope, helps to achieve a high success rate (n = 55; 100%) in the management of difficult per urethral catheterisation. Our proposed algorithm also helps to reduce the need for secondary procedures in most patients, avoids the need for SPC, and, in turn, reduces the cost of hospitalisation and SPC-associated morbidity.

This protocol can be further modified and refined according to the specific requirements of the health facilities and, if implemented properly, can serve as an invaluable resource to manage difficult urethral catheterisation. It will help to reduce the unnecessary burden on the healthcare system by minimising the potential iatrogenic urethral injuries and reducing the need for SPC.
